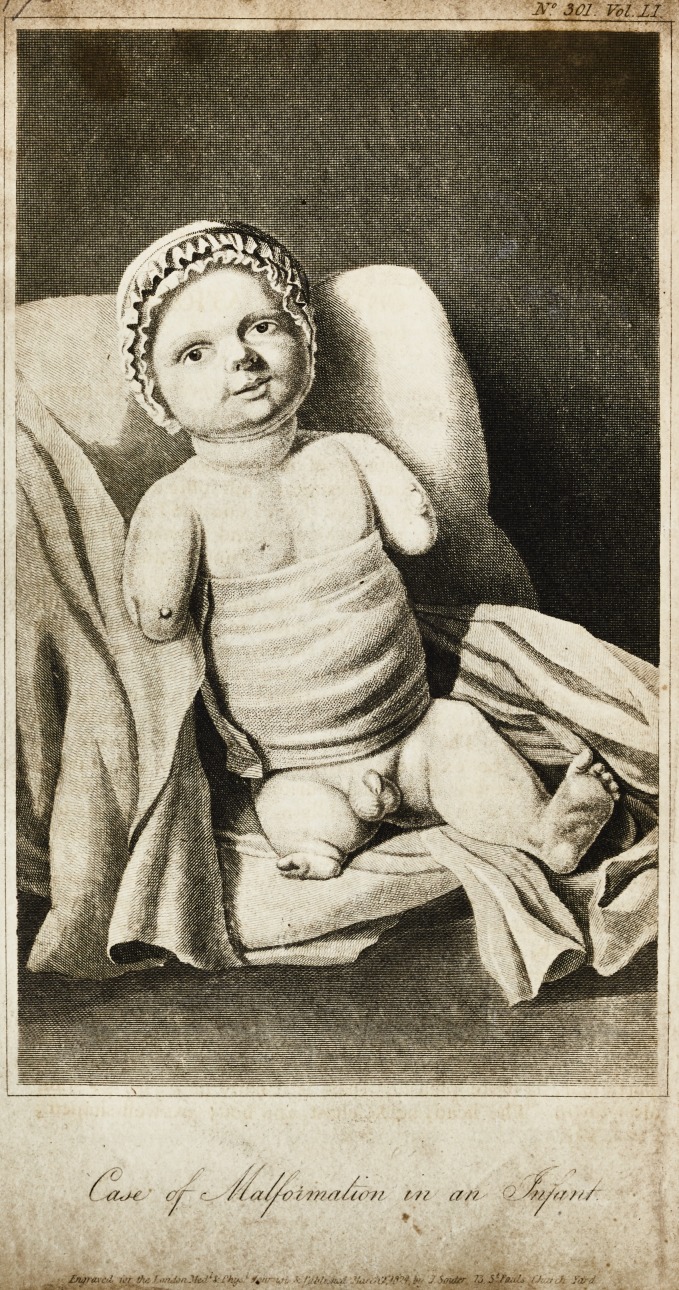# Case of Malformation in an Infant

**Published:** 1824-03

**Authors:** Edward Morton

**Affiliations:** Member of Trinity College, Cambridge, &c.


Case of Malformation in an Infant.

Engraved for the London Medl. & Physl. Journal & Published March 1, 1824, by J. Souter. 73. St. Pauls Church Yard.

THE LONDON

### Medical and Physical Journal.

3 OF VOL. LI.] MARCH, 1824. [NO. 301.

For many fortunate discoveries in medicine, and for the detection ot numerous errors, the world is indebted to the rapid circulation of Monthly Journals; and there never existed any work, to which the Faculty, in Europe and America, were under deeper obligations, than to the Medical and Physical Journal of London, now forming a long, but an invaluable, series.—RUSH.

### ORIGINAL COMMUNICATIONS,

### SELECT OBSERVATIONS, &c.

ART. I.—*Case of Malformation in an Infant.* By EDWARD MORTON, M.B. Member of Trinity College, Cambridge, &c.—[With an Engraving.]

THE following singular instance of malformation in an infant came under my notice a few days ago, while officiating for Dr. Granville; and, at his suggestion, I send you the following account of it for insertion in the Medical and Physical Journal, hoping it may prove not altogether uninteresting to your readers. In order fully to establish the authenticity of the case, I inclose the name and address of the parents; but I refrain from making them public, out of respect to their feelings, lest it might excite idle curiosity, and thereby add to the weight of affliction under which they are at present labouring.

The mother is a patient of the Benevolent Institution, and was attended, during her confinement, by one of the midwives of that charity. The case was a presentation of the breech, and, as might be expected, proved tedious; but no unusual occurrence whatever took place during the progress of the labour. She is a well-formed, middle-aged woman, and has had five labours anteriorly to the present, the first of which produced twins. The children were well-shaped and healthy, and, with the exception of the twins, are now all alive. In her former pregnances, no particularly unpleasant symptoms occurred; but during the last, she says, she has been constantly restless and feverish, and has been much troubled with incontinence of urine. The labour did not occur till the period of utero-gestation was fully completed.

The child (with the exception of those deviations from the natural structure, which have induced me to trouble you upon the present occasion,) is a large, fat, healthy boy: the face, in particular, is more pleasing than is usually met with so soon after birth. The head, neck, chest, and body are well-shaped; but both the superior and inferior extremities are more or less deformed. The arms, after having emerged about three inches from the trunk, terminate in conical extremities, precisely similar in appearance to the stump of an amputated arm. At the end of the right arm there is a small excrescence, like a pimple; while upon the left, at nearly the same place, there is a larger projection, with two smaller eminences upon it, as if nature had here made an imperfect attempt at the formation of a hand and two fingers. The penis is rather larger than is generally found in infants of the same age; the testicles are decidedly larger than usual. All the natural passages are pervious. The left leg and foot are correctly formed, but the thigh is only about one-half its proper length: this defect, of course, brings the knee too near the body, and gives the whole limb an unnatural appearance. The right lower extremity is altogether deformed. The thigh proceeds about two inches from the body, and then abruptly terminates in something like a foot with only two toes.

The cause of this curious malformation is attributed by the mother to a fright, which she experienced when she was about six weeks or two months advanced in pregnancy. The account which she gives is as follows:—She was one day standing at her door, when a beggar without arms, and who had also a wooden leg, came up to her, and demanded some money; she refused to give him any; upon which he suddenly sprung towards her, making a motion with his mutilated arms as if he would embrace her. She was very much frightened at the time, and mentioned it to a neighbour; but did not think of the circumstance again, till she saw the child, when it immediately returned to her recollection.

It may, perhaps, be objected, that the whole of the above statement is a fabrication of the mother, invented after the birth of the child; but, in answer to this, I am enabled to say that, from inquiries which I have instituted, and also from various circumstances, which it would be unnecessary here to detail, I have ascertained, beyond all doubt, that the above account is strictly correct.

This case may possibly, by some, be considered as countenancing the opinion that the mother's imagination possesses the power of deforming the fœtus in utero. I must beg leave to observe, that, from anatomical considerations, I do not believe the possibility of such an occurrence; but still it is worthy of observation, that the period of gestation, at which the fright is stated to have taken place, is precisely the one at which it is probable that the embryo would be most susceptible of such an alteration in its form as the present child exhibits; since it is well known that, at about the seventh week after conception, the extremities of the fœtus are by no means fully formed, but appear to be merely the rudiments of the future limbs.

P.S.—I am indebted to the kindness of a friend for the annexed drawing, which, though taken under every possible disadvantage, presents a faithful representation of the infant.

*15, Eaton-street, Pimlico; Jan. 30th,* 1824.

## Figures and Tables

**Figure f1:**